# Is Youth Smoking Related to the Density and Proximity of Outdoor Tobacco Advertising Near Schools? Evidence from Indonesia

**DOI:** 10.3390/ijerph18052556

**Published:** 2021-03-04

**Authors:** Sri Handayani, Enny Rachmani, Kriswiharsi Kun Saptorini, Yusthin Merianti Manglapy, Nurjanah, Abdillah Ahsan, Dian Kusuma

**Affiliations:** 1 Public Health Program, Faculty of Health Sciences, Universitas Dian Nuswantoro, Semarang 50131, Indonesia; sri.handayani@dsn.dinus.ac.id (S.H.); Yusthin.manglapy@dsn.dinus.ac.id (Y.M.M.); nurjanah@dsn.dinus.ac.id (N.); 2 Health Information Management Program, Faculty of Health Sciences, Universitas Dian Nuswantoro, Semarang 50131, Indonesia; enny.rachmani@dsn.dinus.ac.id (E.R.); kriswiharsi@dsn.dinus.ac.id (K.K.S.); 3 Faculty of Economics and Business, University of Indonesia, Depok 16424, Indonesia; ahsanov@yahoo.com; 4 Centre for Health Economics & Policy Innovation, Imperial College Business School, London SW7 2AZ, UK

**Keywords:** adolescent, smoking, tobacco, advertising, built environment, Indonesia

## Abstract

Background: Smoking was among the top contributors to deaths and disability as the prevalence among male adults remains high, and that among male youth increases in Indonesia. While local studies have shown high visibility of outdoor tobacco advertising around schools, the country still has no outdoor tobacco advertising ban. Objective: To examine the association between youth smoking behavior and measures of outdoor tobacco advertising density and proximity in Indonesia. Methods: We combined two primary data sources, including outdoor tobacco advertising and smoking behavior among male youth in Semarang city. We randomly selected and interviewed 400 male students at 20 high schools in the city. In addition, we interviewed 492 male adults who lived near the schools for comparison. Results: We found significant associations between smoking use among youth (but not among adults) and measures of outdoor tobacco advertising density and proximity in Semarang city. Youth at schools with medium and high density of outdoor tobacco advertising were up to 2.16 times more likely to smoke, compared to those with low density. Similarly, youth at senior high schools with proximity to outdoor tobacco advertising were 2.8 times more likely to smoke. Also, young people at poorer-neighborhood schools with a higher density of and proximity to outdoor tobacco advertising were up to 5.16 times more likely to smoke. Conclusions: There were significant associations between smoking use among male youth (but not among male adults) and measures of outdoor tobacco advertising density and proximity in Indonesia. This highlights the need to introduce an outdoor tobacco advertising ban effectively, at least near schools.

## 1. Introduction

Smoking was among the top contributors to deaths and disability, particularly among men, as shown by the latest Indonesian Global Burden of Study 2017 [1]. The prevalence of smoking among men (15+ years) and boys (13–14 years) was among the highest in the world at 67% (2018) and 36% (2014), respectively [2,3]. Despite all this, the country is still not among the 181 signatories of the Framework Convention of Tobacco Control. In effect, tobacco control efforts are lacking. There is one flagship smoke-free policy that was enacted in 2012 to ban tobacco smoking, advertising, promotion, and sale in selected facilities. However, it has been adopted only by two-thirds of 514 districts by 2018, with the compliance rates ranging from 17% in Jayapura to 78% in Bogor [4,5].

In addition, there is still no national regulation to ban outdoor tobacco advertising. As a consequence, previous studies have shown high visibility of outdoor tobacco advertising around schools in Indonesia. In 2015, a study in five cities found that tobacco billboards were visible from the gate in 32% of 360 sampled high schools [6]. In 2017, a survey of tobacco advertisements and promotions around schools in ten cities (including Semarang) found aggressive marketing strategies by showing brands and very low prices [7]. In 2018, our previous study found a total of 3453 advertisements throughout Semarang city, of which 74% were within a 5–10 min walk from schools [8].

Previous studies from high-income countries have shown that youth are highly receptive to tobacco advertising and that young people exposed to tobacco advertising and promotion are more likely to smoke [9,10,11,12,13]. However, studies on whether outdoor tobacco advertising visibility is associated with smoking behavior among youth is currently lacking in Indonesia and other developing countries [14]. Thus, our study aims to examine the association between youth smoking behavior and measures of outdoor tobacco advertising density and proximity in Indonesia, a lower-middle-income country.

The capital of Central Java province, Semarang city, hosted nearly 1.8 million people in 2018. The city government introduced the smoke-free policy since 2013 but has not been implementing more comprehensive tobacco control measures, including an outdoor tobacco advertising ban. This is partly due to the very high tobacco industry interference from 110 tobacco manufacturers in the province, including PT. Djarum and PT. Gudang Garam, with about 40% of cigarette sales nationally [8].

## 2. Methods

We employed a cross-sectional quantitative study to examine the association between youth smoking behavior and measures of outdoor tobacco advertising density and proximity in Indonesia. We used two primary data sources: outdoor tobacco advertising and smoking behavior among youth and adults in Semarang city. First, the advertising data were from our previous study conducted during November–December 2018 through a survey of outdoor tobacco advertisements. The types of advertisements included billboard, videoboard, banner, store sign, neon box, poster or sticker. There were 3453 outdoor tobacco adverts (including those in front of stores/retailers) with the size ranging from small (between 21 × 30 cm [approximately A4 size]) and 1.3 × 1.9 m) to large (>2.0 × 2.5 m [the size of a typical billboard]). The study also analyzed school data of 978 governmental and private schools in Semarang city, obtained from the city education office on 15 May 2019 (http://disdik.semarangkota.go.id (accessed on 11 December 2020)). In addition to school names and levels (primary, junior high, and senior high), data included addresses that we converted into geocodes using Google Sheets and geocoding add-ons. Further details on methods and results have been published elsewhere [8]. Second, as a follow-up from our previous study [8], we interviewed students of a sample of high schools to observe smoking behavior, defined as ever smoked cigarettes. We randomly selected 20 high schools and interviewed 400 male students (or 20 in each school). For sample calculation, we used Indonesian Global Youth Tobacco Survey (GYTS) data that 50% of youth reported seeing cigarette advertisement or promotion and 5% margin of error, resulting in a minimum sample of 384 students. The inclusion criteria included male, at least 13 years old, at least one year at the junior or senior high school, and willing to be a participant. For comparison, we also interviewed male adults near each school where we randomly selected about 24 adults with the inclusion criteria of male, at least 18 years old, live near the schools, and willing to be a participant.

In this study, we focused on males because of having disproportionately higher smoking prevalence in Indonesia. Smoking prevalence was 10.2% vs. 0.2% among boys and girls (13–14 years old) and 61.4% vs. 2.3% among men and women (15+ years old) in 2018 [15]. In terms of study instruments, we used adaptations of the Global Youth Tobacco Survey (GYTS) and Global Tobacco Adult Survey (GATS) questionnaires from the Ministry of Health (both in the Indonesian language). Data collection was conducted by 11 trained enumerators during September to December 2019. Ethical clearance was obtained from the State University of Semarang (Number: 242/KEPK/EC/2019).

We conducted two types of data analysis: geospatial analysis and quantitative analysis. The geospatial analyses were conducted in ArcMap 10.6. We employed the geoprocessing buffer tool to generate buffers of 200 and 400 m around each school. We used two measures of exposure: density and proximity of advertising. Density was measured by the total number of adverts within 400 m of each school, which we evenly divided into low (0–5 adverts), medium (6–14 adverts), and high (15+ adverts). Proximity was measured by the presence of at least one outdoor tobacco advert within 200 m of school [16,17]. We also used the spatial intersect and join tools to calculate the number of adverts within each buffer. We then matched the exposure results with the binary dependent variable of ever smoking. The quantitative analyses were conducted in STATA 15.1 and employed multiple logit regressions. We produced odds ratios for comparing smoking prevalence between medium-to-low exposure and high-to-low exposure, controlling for age. We also provided subgroup analysis by school level (junior and senior high school) and neighborhood characteristics (poorer and richer areas). For the latter, using data on subdistrict-level poverty rates from the City Statistics Bureau, we used -xtile- command in STATA 15.1 to group the districts (and any schools within) into two groups: poorer and richer areas.

## 3. Results

Table 1 shows descriptive statistics of the youth and adult samples and outdoor tobacco advertising.

We analyzed a total of 892 individuals, including 400 youth and 492 adults. Forty-eight percent of youth were 11–14 years old, and 47% of adults we 18–34 years old. Among the youth sample, 65% and 35% were in junior and senior high schools, respectively. Forty-five percent of youth went to schools, and 45% of adults lived in poorer areas. In terms of outdoor tobacco advertising, only 40% of youth and adults were exposed to a low density of advert, while the other 60% were exposed to medium and high density. In terms of smoking behavior, 65% of the youth sample reported ever smoke cigarettes, while 72% of the adult sample did.

Table 2 shows the association between smoking behavior among youth and adults and measures of outdoor tobacco advert density and proximity.

In terms of density, the odds of smoking among youth were significantly higher up to 2.16 times at schools with medium and high density, compared to that with low density (Table 2 panel a column 1). However, the odds of smoking among adults were not statistically different (Table 2, panel a, column 2). By the school level, the odds of youth smoking were significantly higher up to 2.78 times at junior and senior high schools with high advert density, compared to that with low density (Table 2, panels b–c, column 1). By neighborhood, the odds of youth smoking were significantly higher up to 5.16 times at schools with medium and high density in poorer areas, but not in richer areas (Table 2, panels d–e, column 1). Among adults, the odds of smoking were not statistically different by density tertiles, both in poorer and richer areas (Table 2 panels d–e column 2).

In terms of proximity, the odds of youth smoking were not statistically different between students at schools with at least one advert within 200 m (proximity) and those at schools with no advert within 200 m (Table 2, panel a, column 2). Those odds, however, were significantly higher 2.80 times among students at senior high schools and 2.03 times among students at poorer schools with proximity, compared to otherwise (Table 2, panels b–e, column 2). Among adults, the odds of smoking were not statistically different by proximity both overall and by neighborhood area (Table 2, panels d–e, column 4).

**Figure 1:**
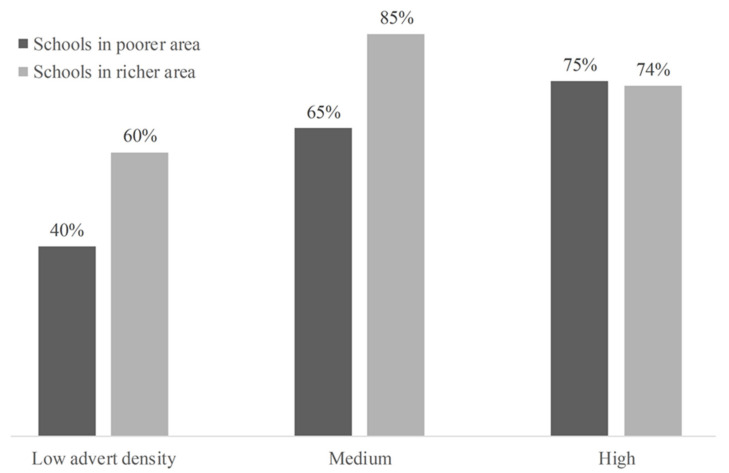
Smoking prevalence among youth and outdoor tobacco advertising density by neighborhood characteristics.

Figure 1 further investigates youth smoking and advert density by neighborhood characteristics.

First, smoking prevalence was higher at schools with medium and high advert density than those with low advert density—the average smoking prevalence was 50% at schools with low density and 75% at those with medium and high density (The results in Table 2 confirmed this). Second, smoking prevalence was higher among students at richer schools, relative to those at poorer schools, but only if the schools had low-to-medium density. The smoking prevalence among students at richer and poorer schools was similar among the schools with high advert density.

## 4. Discussion

Our study showed significant associations between cigarette smoking among youth (but not among adults) and measures of outdoor tobacco advertising density and proximity in Semarang city. Youth at schools with medium and high density of outdoor tobacco advertising were up to 2.16 times more likely to smoke, compared to those with low density. Similarly, youth at senior high schools with proximity (i.e., at least one advert within 200 m) to outdoor tobacco advertising were 2.8 times more likely to smoke. Also, youth at poorer-neighborhood schools with a higher density of and proximity to outdoor tobacco advertising were up to 5.16 times more likely to smoke.

These results align with previous studies from other countries [9,10,11,12,13]. From high-income countries, a Cochrane study reviewed 19 studies in the USA, UK, Germany, and Spain and found that the nonsmoking adolescents who were more aware of tobacco advertising, were more likely to have experimented with cigarettes or become smokers [10]. Further, a study in the United States showed that neighborhoods with the highest proportion of Black or lower-income residents had 2.84 times greater exterior advertisement [18]. From low- and middle-income countries, a study in India showed that smoking use among youth at schools with a high density of outdoor tobacco advertising was more than doubled, compared to those at low density [14].

These findings are significant for at least three reasons. First, there are over 400 high schools in Semarang city alone [8], indicating potential exposure to tobacco advertising for many young people. A study among students in Scotland showed that 80% of nearly 1500 students recalled seeing tobacco advertising at stores [19]. Second, these findings complement our previous study on high outdoor tobacco advertising visibility near schools in the city [8]. This suggests that students are more likely to initiate smoking experimentally either from peer pressure (‘If I don’t smoke, I’m not a real man’) [20] or from encouragement to smoke by advertising [21]. Third, the lack of an outdoor tobacco advertising ban would potentially increase the disparity in youth smoking as our findings show doubling odds ratio among poorer-neighborhood schools. Learning from a study on cigarette retailers in the United States showed that banning tobacco product sales near schools may reduce the density in lower-income neighborhoods compared to higher-income ones [22].

For policy, our findings support for introducing a national outdoor tobacco advertising ban in Indonesia and other developing countries that have not done so. An effective outdoor tobacco advertising ban in Greece has shown to reduce the number of advertising to zero [23], which means elimination of advertising exposure to young people. Hopefully, the government will be able to overcome the huge tobacco company interference and introduce an outdoor tobacco advertising ban, along with other comprehensive MPOWER measures [24]. All this indicates the need to implement the measures of the Framework Convention on Tobacco Control (FCTC) to help reduce or eliminate the tobacco advertising including near schools.

Our study has several limitations. First, due partly to limited funding resource, this current study was conducted one year after the first study on outdoor tobacco advertising locations. We argue that the adverts did not change much as there were no major tobacco efforts in the city during the period. Studies in nearby Banyuwangi city, which was also lacking a total advertising ban, showed similarly higher density of outdoor tobacco adverts around schools between 2018 and 2019 [25,26]. Second, our study was conducted in an urban setting, so findings should not be representative of the whole country. Further study should examine rural districts to explore any regional and socioeconomic variations Also, the study used male sample only, which limits generalization to all gender. Third, our study only interviewed junior and senior high school students. With data showing that smoking initiation is getting younger in the country, further studies should also assess smoking behavior among primary school students. Lastly, our findings identified associations not causations, because of the possible endogenous nature of where outdoor advertising is placed and therefore the endogenous nature of advertising exposure. While these data are consistent with the suggestion that a ban on outdoor advertising might reduce smoking, further study should deal more effectively with the possible endogenous nature of outdoor advertising placement. One could try to examine changes in advertising exposure and changes in smoking behavior for those in the same area. One could also compare youth from the same school but whose commuting path intersects with different levels of outdoor advertising or find some other design (e.g., a diff-in-diff model) that provides a more exogenously determined treatment and control group. Despite all this, our findings have important policy implications for Indonesia and beyond.

## 5. Conclusions

There were significant associations between smoking use among youth (but not among adults) and measures of outdoor tobacco advertising density and proximity in Indonesia. This highlights the need to introduce an outdoor tobacco advertising ban, at least near schools, effectively in Indonesia and beyond. While the findings are consistent with the suggestion that a ban on outdoor advertising might reduce smoking, further study should deal with the possible endogenous nature of outdoor advertising placement.

## Figures and Tables

**Table 1:** Descriptive statistics of youth and adult samples (all males) and outdoor tobacco advertising.

Youth Sample			Adult Sample		
Variable	*n*	%	Variable	*n*	%
	[1]	[2]		[3]	[4]
Total	400			492	
Age group			Age group		
11–14 years	190	48%	18–34 years	232	47%
15–21 years	210	53%	35+ years	260	53%
Level			Neighborhood		
Junior high school	260	65%	Poorer area	223	45%
Senior high school	140	35%	Richer area	269	55%
Neighborhood			Advert density exposure		
Poorer area	180	45%	Low	197	40%
Richer area	220	55%	Medium	147	30%
			High	148	30%
Advert density exposure					
Low	160	40%	Advert proximity exposure		
Medium	120	30%	At least one within 200 m	292	59%
High	120	30%	No advert within 200 m	200	41%
Advert proximity exposure			Smoking status		
At least one within 200 m	240	60%	Ever smoke cigarette	354	72%
No advert within 200 m	160	40%	Otherwise	138	28%
Smoking status					
Ever smoke cigarette	258	65%			
Otherwise	142	36%			

Note: *n* = sample, % = proportion. There were 400 students interviewed from 20 high schools (so 20 students per school). Out of 400 youth samples, 382 students were 11–17 years old, 14 students were 18 years old, and 4 students were 19 or 21 years old. There were 492 adults interviewed near those 20 high schools (24–25 adults per school). For neighborhood, poorer/richer areas are with higher/lower subdistrict-level poverty rates. The measures of density and proximity were the same for youth/students and adult samples. Density was measured by the total number of adverts within 400 m of each school. For density, basic descriptive = mean 9.75, standard deviation 7.97, and range 0–24.

**Table 2:** Association between smoking behavior among youth and adults and measures of outdoor tobacco advert density and proximity in Semarang, Indonesia.

	Youth Sample				Adult Sample			
	Density		Proximity		Density		Proximity	
	OR (SE)		OR (SE)		OR (SE)		OR (SE)	
	[1]		[2]		[3]		[4]	
(a) Overall	*n* = 400				*n* = 492			
Density tertiles								
Low	Ref				Ref			
Medium	1.93 **	(0.52)			1.25	(0.31)		
High	2.16 **	(0.59)			1.01	(0.24)		
Proximity								
At least one within 200 m			0.97	(0.22)			0.95	(0.20)
(b) Junior high schools	*n* = 260							
Density tertiles								
Low	Ref					NA		
Medium	1.76	(0.54)						
High	1.93 **	(0.64)						
Proximity								
At least one within 200 m			0.68	(0.18)				
(c) Senior high schools	*n* = 140							
Density tertiles								
Low	Ref					NA		
Medium	2.83	(1.58)						
High	2.78 **	(1.38)						
Proximity								
At least one within 200 m			2.80 **	(1.23)				
(d) Poorer areas	*n* = 180				*n* = 223			
Density tertiles								
Low	Ref				Ref			
Medium	2.64 **	(0.89)			1.66	(0.55)		
High	5.16 **	(3.00)			0.96	(0.46)		
Proximity								
At least one within 200 m			2.03 **	(0.64)			1.70	(0.53)
(e) Richer areas	*n* = 220				*n* = 269			
Density tertiles								
Low	Ref				Ref			
Medium	0.99	(0.60)			0.79	(0.30)		
High	1.01	(0.37)			0.94	(0.29)		
Proximity								
At least one within 200 m			0.19 **	(0.08)			0.55	(0.18)

Note: OR = Odds Ratio, SE = Standard Errors, *n* = Sample, Ref = Reference group. Odds ratios were obtained from logit regressions of smoking status on density/proximity, controlling for age (in STATA 15.1). There were 400 students interviewed from 20 high schools; 492 adults interviewed near those schools. The measures of density and proximity were the same for youth and adults. Density was measured by the total number of adverts within 400 m of each school. For neighborhood, poorer/richer areas are with higher/lower subdistrict-level poverty rates. The density was 1.43 times higher at schools in richer (mean = 11.27, SD = 8.29) areas than those in poorer areas (mean = 7.89, SD = 7.14). ** = significant at 5% level.

## Data Availability

Available upon reasonable request.
